# Synthesis of Two New Hemisynthetic Diterpenylhydroquinones from Natural *Ent*-Labdanes

**DOI:** 10.3390/molecules14062181

**Published:** 2009-06-17

**Authors:** Luis Espinoza Catalán, Karen Catalán Marín, Alejandro Madrid Villegas, Héctor Carrasco Altamirano, Joan Villena García, Mauricio Cuellar Fritis

**Affiliations:** 1 Departamento de Química, Universidad Técnica Federico Santa María, Av. España N° 1680, Valparaíso, Chile; 2 Departamento de Ciencias Químicas, Universidad Andrés Bello, Campus Viña del Mar, Los Fresnos N° 52, Viña del Mar, Chile; 3 Facultad de Medicina, Universidad de Valparaíso, Centro Regional de Estudios en Alimentos Saludables, Creas, Av. Hontaneda N° 2664, Valparaíso, Chile; 4 Facultad de Farmacia, Universidad de Valparaíso, Av. Gran Bretaña N° 1093, Valparaíso, Chile

**Keywords:** diterpenyl-hydroquinones, synthesis, *ent*-labdanes, NMR structural determination

## Abstract

The synthesis and structural determination of two new diterpenylhydroquinones: 2β-acetoxy-15-phenyl-(22,25-dihydroxy)-*ent*-labda-8(17),13(*E*)-diene (**1**) and 2β-hydroxy-15-phenyl-(22,25-dihydroxy)-*ent*-labda-8(17),13(*E*)-diene is reported (**2**). These compounds were obtained by coupling via Electrophilic Aromatic Substitution (*EAS*) of 1,4-hydroquinone with primary or tertiary allyl alcohol derivatives of the natural *ent*-labdanes **3** and **4**. With this new method, the best results were observed when mixtures of the primary alcohol derivatives **5**-**6** (26% yield of compound **1**) and diol derivatives **9**-**10** (28% yield of compound **2**) were used.

## Introduction

Terpenylquinones and terpenylhydroquinones are characteristic marine metabolites with examples of 4,9-friedodrimane, drimane and nordrimane skeletons, frequently isolated from alga and/or sponge genera [1]. This class of compounds has attracted the attention of researchers because to their potent biological properties, which include antimicrobial [2], antileukemic [3], cytotoxic [4,5,6], hemolytic [7], and immunomodulatory activities [8,9]. In the late 1980s, there was significant interest in the possible anti-HIV activity of marine sesquiterpenes [1,10,11,12]. These compounds are generally characterized as having a bicyclic sesquiterpene skeleton attached to a (hydro)quinone moiety in its structures.

**Figure 1:**
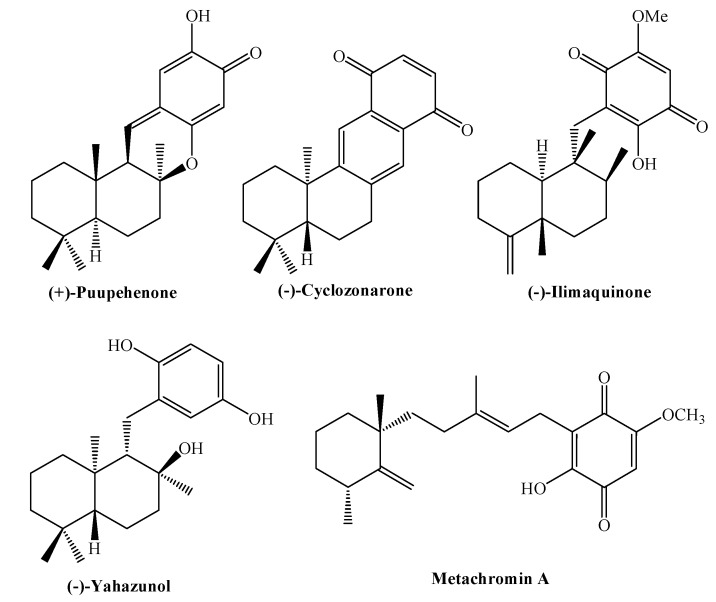
Structure of some natural terpenyl-(hydro)quinones.

The more recurrent synthetic strategies used for synthesizing terpenylquinones/hydroquinones, involve, as a first step, the separate preparation of the appropriate terpenyl and aromatic nucleus fragments. The crucial step is the attachment of the aromatic synthon to the terpenyl skeleton and the final step is the formation of oxygenated functions (generally by oxidation of hydroxyl or methoxyl groups) on the quinone moiety. The more used methods for the coupling reactions are the following: 1) Nucleophilic addition of aryllithium derivatives to carbonyls of the terpenyl unit (synthesis of (+)-puupehenone [13]); 2) Diels-Alder and hetero Diels-Alder cycloaddition (synthesis of (-)-cyclozonarone [14] and (+)-cyclozonarone [15]); 3) Enolate alkylation with benzyl bromide derivatives (synthesis of (-)-ilimaquinone [16]); 4; Conjugated 1,4-addition; Michael addition (synthesis of (-)-yahazunol [17]); 5) Coupling of Horner-Wadsworth-Emmons (HWE) (synthesis of metachromin **A** [18]). All these structures are shown in Figure 1.

**Scheme 1:**
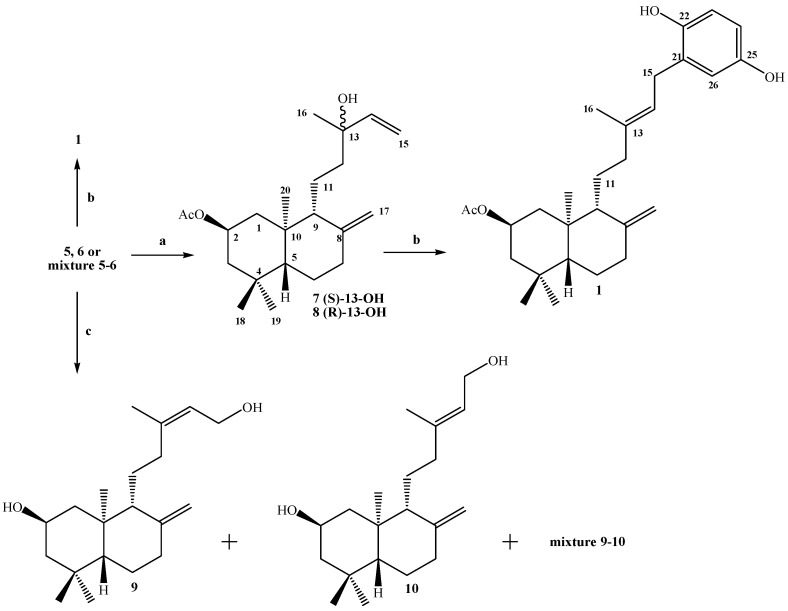
Synthesis of compound **1**.

**Scheme 2:**
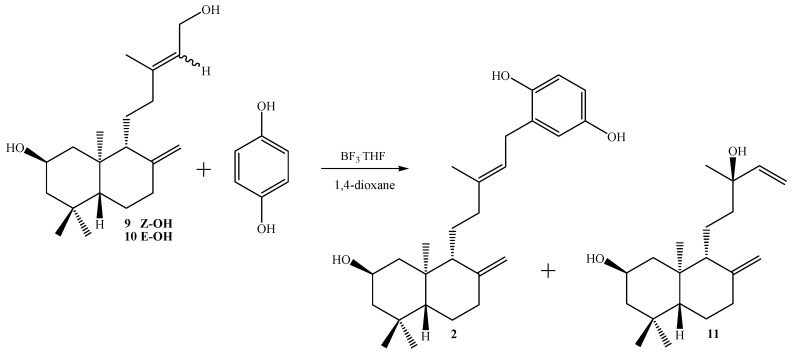
Synthesis of compounds **2** and **11**.

In this work we report the first synthesis and the structural determination of two new bicyclic diterpenyl-hydroquinones **1** and **2** (Scheme 1 and Scheme 2) with *ent*-labdane skeletons and a bridge between the terpenyl unit and hydroquinonic nuclei consisting of five carbon atoms and an (*E*)-trisubstituted double bond, as found in the structure of metachromin **A**.

**Figure 2:**
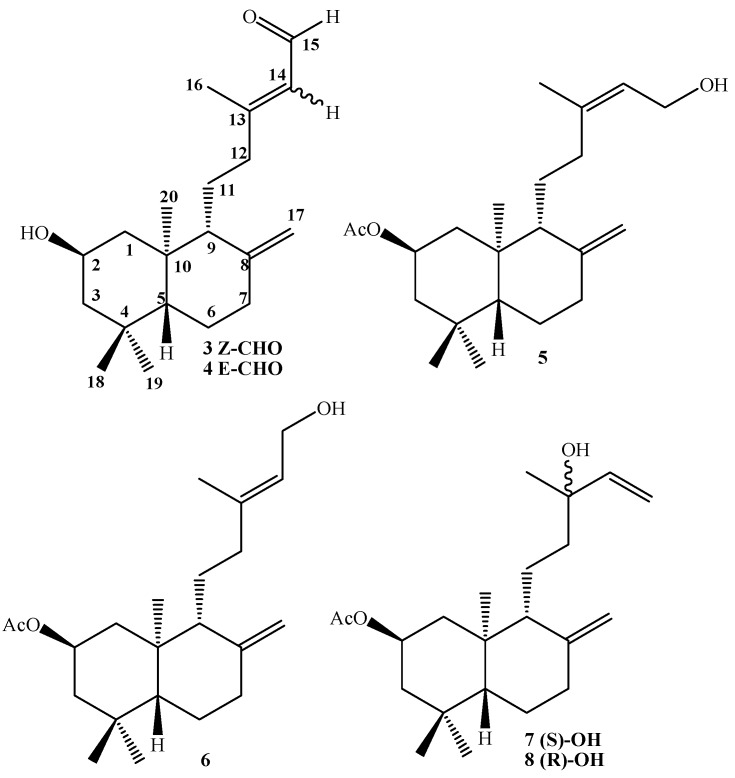
Structure of natural ent-labdanes and derivatives.

For the coupling reaction (key step of the synthesis) between both fragments, a strategy involving Electrophilic Aromatic Substitution (EAS) of diterpenyl allylic alcohol derivatives **5**-**8** (Figure 2) and diols **9**-**10** with 1,4-hydroquinone were used, as was described for the synthesis of a taondiol derivative [19,20], although it is important to emphasize that the use of an EAS reaction as the like key coupling reaction step has not been previously reported for the syntheses of terpenylquinones. The results of the biological activities against cancer cellular lines of these compounds will be reported in complete detail elsewhere at a later date.

## Results and Discussion

In previous investigations we reported the isolation and structural determination of the mixture of *ent*-labdanes **3**-**4** from *Calceolaria inamoena* and the preparation of derivatives **5** and **6** (Figure 2) [22]. Our next step was the preparation of the tertiary alcohol **7** and/or **8**, from primary allyl alcohols **5** or **6**. Nevertheless, when these compounds or a mixture of both, were reacted with SOCl_2_, in the three cases small amounts of the epimeric mixture **7**-**8** was obtained (see Scheme 1) along with a complex unidentified mixture of compounds which was obtained as the major component. The highest yield of **7**-**8** mixture (33.7%) was observed when compound **6** was treated with SOCl_2_. Unfortunately this mixture could not be separated by conventional chromatography and the proportion of epimers in the mixture were determined as **7**:**8** =0.8:1 (ratio calculated based on the integrals of the Me-20 signals in the ^1^H-NMR spectrum). The stereochemistry determination for the C-13 position was proposed by comparison and correlations with *δ*^13^C data of both epimers (δ_C_ = 73.4 for **7** and δ_C_ = 73.6 ppm for **8**) with those reported for other related compounds [22,23], such as (-)-*S*-sclareol, (+)-*R*-sclareol, (-)-*R*-manool and (+)-*S*-manool, where the C-13 of the 13-(*R*) epimer always showed a greater chemical shift than the corresponding 13-(*S*) epimer.

*Conditions and reagents*: **a**. From **5**: SOCl_2_/CH_2_Cl_2_/py, N_2_, T=-10°C, 1 h, 22.1%; from **6**: 33.7%; from **5**-**6**: 30.3%. **b**. From **5**-**6**: 1,4-hydroquinone/1,4-dioxane/BF_3_^.^THF, N_2_, T=20°C, 24 h, 11%; from **5**: 10% and from **6**: 20%. **c**. From **5**-**6**: K_2_CO_3_/MeOH, r.t, 0.5 h, C.C., **9**, 11.3%; **9**-**10**, 20.4% and **10**, 55.4%.

**Figure 3:**
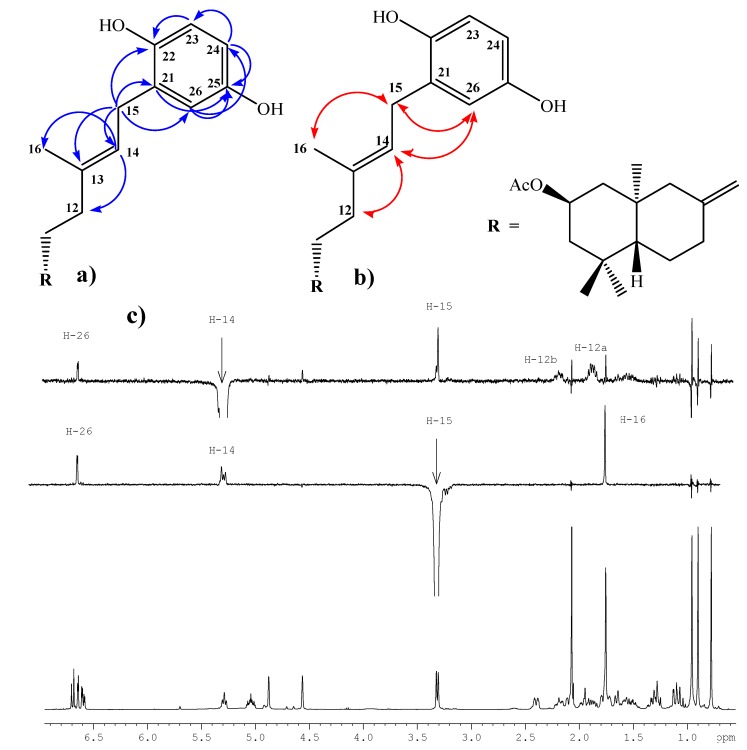
Structure of compound **1**. (a) HMBC correlations. (b) NOE correlations. (c) bottom: normal ^1^H-NMR spectrum, middle: selective irradiation at = 3.18 ppm, top: selective irradiation at = 5.23 ppm (the sel. gs. 1D ^1^H-NOESY spectra were registered using *selnogp.3* Bruker pulse program and parameter set: ns = 32, p12 = 80 ms and d8 = 400 ms).

The next step was the coupling reaction between the **7**-**8** mixture with 1,4-hydroquinone catalyzed by BF_3_^.^THF, according to the previously described protocol [19,20]. After the corresponding workup and purification of the crude product by column chromatography (CC), only two major products were isolated: 0.678 g of an unidentified complex mixture (non-polar fraction) and 0.127 g (11%) of the desired compound **1**. The structural determination of compound **1** was mainly accomplished by ^1^H-, ^13^C-, DEPT-135, gs-2D HSQC and gs-2D HMBC NMR techniques. In its IR spectrum absorptions at 3,401 and 1,701 cm^-1^ were observed, whereas the ^1^H-NMR spectrum showed the existence of three aromatic hydrogens at δ = 6.67 (d, *J*= 8.6 Hz, 1H, H-23); 6.62 (d, *J*= 3.0 Hz, 1H, H-26) and 6.57 (dd, *J*= 8.6 and 3.0 Hz, 1H, H-24) and in the ^13^C-NMR spectrum the presence of six aromatic carbons was also observed. In addition, the signal at 3.29 ppm (d, *J*= 7.1 Hz, 2H, H-15) correlated (by 2D HSQC) with a carbon atom at δ 29.3 ppm (C-15), indicating the coupling point between the diterpenyl fragment and the aromatic nucleus. These data also were corroborated by 2D HMBC correlations, where H-15 showed heteronuclear *^3^J* correlations with the carbon signals at δ 116.5 (C-26), 138.5 (C-13) and 147.8 (C-22) ppm. Heteronuclear *^2^J* correlations also were observed at δ 121.3 (C-14) and 128.3 (C-21), these and some 2D HMBC important correlations are shown in Figure 3a. 

The *E*-geometrical spatial orientation of the trisubstituted double bond in C13-C14, was deduced from gs-sel-^1^H 1D-NOESY experiments: when H-15 was selectively irradiated, long range interactions (strong) with the Me-16 group (1.73 ppm) and H-26 (6.62 ppm) were observed, whereas H-14 showed long range interactions (medium) with H-26, H-12a (1.84 ppm) and H-12b (2.17 ppm) (see figure 3b and 3c).

Additionally we decided to try the coupling reaction with 1,4-hydroquinone separately, using the compounds **5**, **6** and a mixture of both (see Scheme 1), following the previously protocol described for the **7**-**8** mixture. In the case of alcohol **5** (1.39 g), after usual workup followed of CC separation, three fractions were obtained: 1.33 g of a non-identified complex mixture (non-polar fraction), 0.175 g (10% yield) of compound **1** and 0.37 g of unreacted 1,4-hydroqinone. In the case of alcohol **6** (1.54 g) were obtained: 0.983 g of complex mixture, 0.390 g (20% yield) of compound **1** and 0.28 g of unreacted 1,4-hydroquinone. In the case of a **5**-**6** mixture (3.92 g, approx. ratio **5**:**6** = 0.25:0.75) 2.47 g of complex mixture, 1.29 g ( 26% yield) of compound **1** and 0.963 g of unreacted 1,4-hydroquinone were obtained. The characterization of compound **1** was accomplished by simple inspection and comparison of ^1^H- and ^13^C-NMR spectral data with that previously obtained.

We also performed the coupling reaction using the diols **9**, **10** and a mixture of both (see Scheme 1) following the experimental procedure previously described. The diols were obtained by alkaline hydrolysis with K_2_CO_3_/MeOH, acidification and later separation and purification by CC. From 2.93 g of the acetate mixture **5**-**6**, 0.292 g of diol **9** (11.3% yield), 0.527 g (yield 20.4%) of a **9**-**10** mixture and 1.43 g (55.4% yield) of diol **10** were obtained. The structures of compounds **9** and **10** were mainly established by ^1^H- and ^13^C-NMR spectroscopic data (see Experimental section) and compared with those reported for acetates **5** and **6,** respectively [21]. The proportion of geometrical isomers in the mixture were determined as **9**:**10** =0.2:1 (ratio calculated based on the integrals of the H-17a and H-17b signals in the ^1^H-NMR spectrum). When compound **9** (0.238 g) was reacted with 1,4-hydroquinone, a non polar unidentified complex mixture (0.183 g) and 40 mg (13%) of compound **2** were obtained. From the compound **10** (0.897 g) 0.420 g of complex mixture and 0.246 g (21%) of compound **2** were obtained. On the other hand, from **9**-**10** mixture (0.258 g) 0.122 g of the complex mixture, 57 mg (17%) of diol **11** and 94 mg (28%) of compound **2** were isolated (see Scheme 2).

*Conditions*: From **9**: N_2_, T=20°C, 24 h, 13%; from **10**: 21%; and from **9**-**10** mixture: **11**, 17% and **2**, 28%.

The structural determination of compound **2** was established by comparison of the spectral data of compound **1** and using the same criteria. In the IR spectrum of compound **2** a strong absorption at 3,375 cm^-1^ was mainly observed, whereas the ^1^H-NMR spectrum showed the existence of three aromatic hydrogens at δ = 6.67 (d, *J*= 8.6 Hz, 1H, H-23); 6.64 (d, *J*= 2.9 Hz, 1H, H-26) and 6.57 (dd, *J*= 8.6 and 2.9 Hz, 1H, H-24), while in the ^13^C-NMR spectrum the presence of six aromatic carbons also was observed. In addition the signal at 3.29 ppm (d, *J*= 7.1 Hz, 2H, H-15) correlated (by 2D HSQC) with a carbon atom at δ 29.0 ppm (C-15), indicating the coupling point between the diterpenyl fragment and the aromatic nucleus. These data also were corroborated by 2D HMBC correlations, where H-15 showed heteronuclear *^3^J* correlations with the carbons signal at δ 116.6 (C-26), 138.1 (C-13) and 147.7 (C-22) ppm. Heteronuclear *^2^J* correlations were also observed at δ 121.7 (C-14) and 128.6 (C-21).

The structure of compound **11** was established by spectroscopic correlations of its ^1^H- and ^13^C-NMR data with the **7**-**8** mixture and the stereochemistry in C-13 position was tentatively assigned as “*R*” by comparison and correlations with *δ*^13^C data of epimers **7** and **8**, which showed a chemical shift at δ_C_ = 73.5 ppm for this carbon.

**Figure 4:**
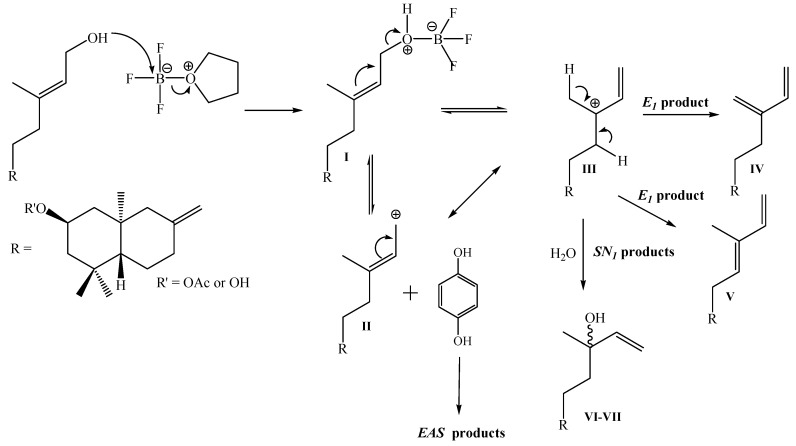
General schemes with probable intermediaries of reaction **II** and **III** and *E_1_* elimination products, which would be competing with the desired coupling reaction via *EAS*

As previously indicated in all the coupling reactions, the formation of a complex mixture of products was observed. Nevertheless by simple inspection of the ^1^H- and ^13^C-NMR spectra of this mixture, we observed the presence of hydrogen (δ_H_: 6.45-6.14 ppm) and carbon (δ_C_: 150.6-142.4 ppm) vinyl signals that were probably due to the formation of mixtures of alkenes (**IV** and **V**), presumably obtained via formation of a cationic intermediary **III** (formed by reaction of the allyl alcohols with BF_3_^.^THF, followed by rearrangement of intermediate **I** and/or **II**), later elimination *E_1_* products or in one case *SN_1_* (for example the formation of compound **11**), that compete with the formation of Electrophilic Aromatic Substitution (EAS) products (see Figure 4)

## Conclusions

In this work we have described the synthesis and structural determination of two new diterpenyl-hydroquinones from natural *ent*-labdanes. These compounds were obtained by coupling primary or tertiary allyl alcohol *ent*-labdane derivatives with 1,4-hydroquinone by Electrophilic Aromatic Substitution (*EAS*). This method of coupling between terpenyl fragments and aromatic nucleus (key step in all synthesis of terpenyl-(hydro)quinones) had not been previously described, and worked for all the allylic alcohols used here. Nevertheless, the best results were observed when the mixtures of primary alcohols **5**-**6** (26% yield of compound **1**) and mixture of diols **9**-**10** (28% yield of compound **2**) were used for coupling reaction. We think that the possible formation of very stable intermediary **III** (Figure 4) is mainly responsible for the formation of a complex mixture of alkenes and the low yields observed in the coupling reactions.

## Experimental

### General

Unless otherwise stated, all chemical reagents purchased (Merck or Aldrich) were of the highest commercially available purity and were used without previous purification. Melting points were measured (in triplicate) on a Stuart-Scientific SMP3 apparatus and are uncorrected. IR spectra were recorded as thin films in a Nicolet Impact 420 spectrometer and frequencies are reported in cm^-1^. Optical rotations were measured with a sodium lamp (λ=589 nm, D line) on a Perkin Elmer 241 digital polarimeter equipped with 1 dm cells at the temperature indicated in each case. Low resolution mass spectra were recorded on a Shimadzu QP-2000 spectrometer at 70eV ionising voltage and are given as m/z (% rel. int.) ^1^H-, ^13^C- (DEPT 135 and DEPT 90), sel. 1D ^1^H NOESY, sel. 1D ^1^H TOCSY, 2D HSQC and 2D HMBC spectra were recorded in CDCl_3_ solutions and are referenced to the residual peaks of CHCl_3_ at δ 7.26 ppm and δ 77.0 ppm for ^1^H and ^13^C, respectively, on a Bruker Avance 400 Digital NMR spectrometer, operating at 400.1MHz for ^1^H and 100.6MHz for ^13^C. Chemical shifts are reported in δ ppm and coupling constants (*J*) are given in Hz. Silica gel (Merck 200-300 mesh) was used for C.C. and silica gel plates HF-254 for TLC. TLC spots were detected by heating after spraying with 25% H_2_SO_4_ in H_2_O.

*Synthesis of 2*β*-acetoxy-(S)-13-hydroxy-ent-labda-8(17), 14-diene* (**7**) *and 2*β*-acetoxy-(R)-13-hydroxy-ent-labda-8(17), 14-diene* (**8**) *from* **5**: A solution of **5** (1.54 g, 0.44 mmol) in dry CH_2_Cl_2_ (50 mL) and dry pyridine (1 mL), was prepared under a N_2_ atmosphere and cooled to -10°C (ice/acetone/brine bath). Then SOCl_2 _(0.6 mL, 8.26 mmol) was slowly added dropwise while maintaining slow agitation. After one hour, the completion of the reaction were verified by TLC. Then saturated aqueous solution of NaHCO_3_ (50 mL) was added and the mixture was extracted with EtOAc (2 x 25 mL) and the combined organic layers were washed with water (2 x 20 mL), dried over Na_2_SO_4_, filtered and evaporated. The crude was redissolved in CH_2_Cl_2_ (5 mL) and chromatographed on silica-gel with petroleum ether/EtOAc mixtures of increasing polarity (19.8:0.2→8.8:11.2). Two fractions were obtained: Fraction I: 1.08 g of a non-polar unidentified complex mixture (colorless viscous oil) Fraction II: colorless viscous oil, 0.34 g (22.1%) of a mixture of **7**-**8**. 

*Synthesis of* **7**-**8** *mixture from* **6**: From 2.23 g (6.40 mmol) of alcohol **6**, 0.8 mL (11.0 mmol) of SOCl_2 _and 1.5 mL of pyridine, 1.32 g of non-polar complex mixture and 0.75 g (33.7%) of **7**-**8** mixture were obtained.

*Synthesis of* **7**-**8** *mixture from a mixture of* **5-6**: From 2.38 g (6.83 mmol) of **5**-**6** mixture, 0.8 mL (11.0 mmol) of SOCl_2_ and 1.5 mL of pyridine, 1.57 g of non-polar complex mixture and 0.72 g (30.3%) of **7**-**8** mixture were obtained. 

Compound **7**: ^1^H-NMR: 5.90 (dd, *J*= 17.3 and 10.8 Hz, 1H, H-14); 5.20 (dd, *J*= 17.3 and 1.5 Hz, 1H, H-15b); 5.05 (dd, *J*= 10.8 and 1.5 Hz, 1H, H-15a); 5.02 (ddt, *J*= 11.7, 11.7 and 3.9 Hz, 1H, H-2); 4.89 (s, 1H, H-17b); 4.55 (s, 1H, H-17a); 2.39 (ddd, *J*= 12.7, 4.4 and 2.5 Hz, 1H, H-7α); 2.07 (m, 1H, H-1α); 2.03 (s, 3H, CH_3_CO_2_); 1.95 (ddd, *J*= 13.2, 12.7 and 4.9 Hz, 1H, H-7β); 1.70 (m, 3H, H-3α, H-12b and H-6β); 1.61 (bd, *J*= 10.3 Hz, 1H, H-9); 1.43 (m, 1H, H-11b); 1.32 (m, 1H, H-11a); 1.29 (m, 2H, H-12a and H-6α); 1.27 (s, 3H, H-16); 1.23 (m, 1H, H-3β); 1.09 (dd, *J*= 17.7 and 2.5 Hz, 1H, H-5); 1.07 (dd, *J*= 11.7 and 11.7 Hz, 1H, H-1β), 0.93 (s, 3H, H-18); 0.88 (s, 3H, H-19); 0.76 (s, 3H, H-20); ^13^C-NMR: 44.1 (C-1), 69.3 (C-2), 46.8 (C-3), 34.9 (C-4), 55.0 (C-5), 23.9 (C-6), 38.0 (C-7), 147.4 (C-8), 57.1 (C-9), 41.2 (C-10), 17.9 (C-11), 41.1 (C-12), 73.4 (C-13), 145.2 (C-14), 111.7 (C-15), 27.5 (C-16). 107.6 (C-17), 33.6 (C-18), 22.4 (C-19), 15.2 (C-20), 170.6 (CH_3_CO), 21.5 (CH_3_CO); M.S.(m/z, %): M^+^ 348 (< 1%), 202 (13.2); 201 (13.9); 189 (13.9); 188 (20.8); 187 (33.0); 175 (16.0); 173 (8.9); 161 (10.5); 159 (12.8); 147 (11.4); 136 (14.5); 135 (100); 134 (15.9); 133 (17.2); 131 (12.2); 122 (10.5); 121 (27.1); 120 (15.0); 119 (28.0); 109 (13.8); 107 (32.3); 105 (14.8); 95 (18.3); 94 (9.4); 93 (29.9); 91 (18.1); 81 (21.1); 80 (8.9); 79 (18.0); 71 (22.5). 

Compound **8**: ^1^H-NMR: 5.90 (dd, *J*= 17.4 and 10.8 Hz, 1H, H-14); 5.21 (dd, *J*= 17.4 and 1.0 Hz, 1H, H-15α); 5.06 (dd, *J*= 10.8 and 1.0 Hz, 1H, H-15α); 5.02 (ddt, *J*= 11.7, 11.7 and 3.9 Hz, 1H, H-2); 4.84 (s, 1H, H-17b); 4.50 (s, 1H, H-17α); 2.39 (ddd, *J*= 12.7, 4.4 and 2.5 Hz, 1H, H-7a); 2.07 (m, 1H, H-1a); 2.03 (s, 3H, CH_3_CO_2_); 1.95 (ddd, *J*= 13.2, 12.7 and 4.9 Hz, 1H, H-7β); 1.70 (m, 3H, H-3α, H-12b and H-6β); 1.61 (bd, *J*= 10.3 Hz, 1H, H-9); 1.43 (m, 1H, H-11b); 1.32 (m, 1H, H-11a); 1.27 (m, 2H, H-12a and H-6β); 1.27 (s, 3H, H-16); 1.23 (m, 1H, H-3β); 1.09 (dd, *J*= 17.7 and 2.5 Hz, 1H, H-5); 1.07 (dd, *J*= 11.7 and 11.7 Hz, 1H, H-1β), 1.04 (s, 3H, H-18); 0.88 (s, 3H, H-19); 0.75 (s, 3H, H-20). ^13^C-NMR: 44.2 (C-1), 69.3 (C-2), 46.8 (C-3), 34.9 (C-4), 55.0 (C-5), 23.9 (C-6), 38.0 (C-7), 147.5 (C-8), 57.0 (C-9), 41.1 (C-10), 17.8 (C-11), 41.2 (C-12), 73.6 (C-13), 145.0 (C-14), 111.8 (C-15), 28.3 (C-16). 107.4 (C-17), 33.6 (C-18), 22.4 (C-19), 15.2 (C-20), 170.6 (CH_3_CO), 21.5 (CH_3_CO). M.S. (m/z, %): M^+^ 348 (< 1%), 302 (4.9); 270 (10.3); 255 (28.2); 136 (14.8); 135 (100); 134 (16.8); 133 (17); 122 (10.9); 121 (26.9); 120 (14.7); 119 (27.3); 109 (13.2); 107 (31.6); 105 (21.6); 95 (18.1); 93 (29.0); 91 (17.7); 81 (20.2); 79 (17.6); 71 (22.0). Mixture **7**-**8**, IR (cm^-1^): 3,483, 3,069, 2,960, 1,736, 1,721, 1,644, 1,470, 1,368, 1,255, 1,209.

*Synthesis of 2β-acetoxy-15-phenyl-(22,25-dihydroxy)-ent-labda-8(17),13(E)-diene* (**1**) *from* **7**-**8*** mixture:* To a solution of 1,4-hydroquinone (0.30 g, 2.27 mmol) and BF_3_^.^THF (0.5 mL, 3.98 mmol) in freshly distilled 1,4-dioxane (5 mL) was slowly added dropwise, with stirring at room temperature and under a N_2_ atmosphere, a solution of **7**-**8** mixture (0.91 g, 2.61 mmol) in 1,4-dioxane (5 mL). After the addition was complete, stirring at room temperature and under N_2_ atmosphere was continued overnight. When the completion of the reaction was verified by TLC, the mixture was poured onto crushed ice (app. 30 g) and the organic layer extracted with diethyl ether (3 x 30 mL), the ethereal layer was washed with 5% NaHCO_3_ (30 mL), then with water (2 x 20 mL) and dried over Na_2_SO_4_, filtered and evaporated. The crude was redissolved in CH_2_Cl_2_ (5 mL) and chromatographed on silica-gel with petroleum ether/EtOAc mixtures of increasing polarity (19.8:0.2→11.4:8.6). Three fractions were obtained: Fraction I: 0.678 g of non-polar non-identified complex mixture (colorless viscous oil) Fraction II: colorless viscous oil, 0.127 g (11%) of compound **1**, and Fraction III: 8.0 mg of unreacted 1,4-hydroquinone.

*Synthesis of* **1** *from* **5**: 1,4-hydroquinone (0.50 g, 4.54 mmol), BF_3_^.^THF (0.5 mL, 3.98 mmol) and **5** (1.39 g, 3.96 mmol) were used. After workup and CC purification: 1.33 g of complex mixture, 0.175 g (10%) of **1** and 0.37 g of unreacted 1,4-hydroquinone were obtained.

*Synthesis* **1** *from* **6**: 1,4-hydroquinone (0.50 g, 4.54 mmol), BF_3_^.^THF (0.7 mL, 5.59 mmol) and **6** (1.54 g, 4.42 mmol) were used. After workup and CC purification: 0.983 g of complex mixture, 0.39 g (20%) of **1** and 0.28 g of unreacted 1,4-hydroqinone, were obtained.

*Synthesis* **1** *from* **5**-**6** *mixture:* 1,4-hydroquinone (1.30 g, 11.8 mmol), BF_3_^.^THF (1.5 mL, 11.9 mmol) and **5**-**6** mixture (3.92 g, 11.25 mmol) were used. After workup and CC purification: 2.47 g of complex mixture, 1.29 g (26%) of **1** and 0.963 g of unreacted 1,4-hydroqinone, were obtained.

Compound **1**, colorless viscous oil, [α]_D_^23^ = -2.6º (c 1.85, CHCl_3_); ^1^H-NMR: 6.67 (d, *J*= 8.6 Hz, 1H, H-23); 6.62 (d, *J*= 3.0 Hz, 1H, H-26); 6.57 (dd, *J*= 8.6 and 3.0 Hz, 1H, H-24); 5.26 (t, *J*= 7.1 Hz, 1H, H-14); 5.03 (ddt, *J*= 12.0, 12.0 and 4.2 Hz, 1H, H-2); 4.85 (s, 1H, H-17b); 4.54 (s, 1H, H-17a); 3.29 (d, *J*= 7.1 Hz, 2H, H-15); 2.38 (ddd, *J*= 13.0, 4.4 and 2.5 Hz, 1H, H-7α); 2.17 (ddd, *J*= 13.9, 8.8 and 4.4 Hz, 1H, H-12b); 2.06 (m, 1H, H-1α); 2.05 (s, 3H, CH_3_CO_2_); 1.92 (ddd, *J*= 13.0, 12.5 and 4.4 Hz, 1H, H-7β); 1.84 (m, 1H, H-12a); 1.75 (m, 1H, H-3α); 1.73 (s, 3H, H-16); 1.71 (m, 1H, H-6β); 1.63 (bd, *J*= 10.3 Hz, 1H, H-9); 1.51 (m, 2H, H-11a and H-11b); 1.29 (dd, *J*= 13.2 and 3.9 Hz, 1H, H-6α); 1.26 (dd, *J*= 12.0 and 12.0 Hz, 1H, H-3β); 1.09 (dd, *J*=13.2 and 2.5 Hz, 1H, H-5); 1.05 (dd, *J*= 12.0 and 12.0 Hz, 1H, H-1β); 0.93 (s, 3H, H-18); 0.88 (s, 3H, H-19); 0.75 (s, 3H, H-20). ^13^C-NMR: 44.0 (C-1), 69.8 (C-2), 46.7 (C-3), 34.9 (C-4), 54.8 (C-5), 23.8 (C-6), 37.9 (C-7), 147.4 (C-8), 55.8 (C-9), 40.9 (C-10), 22.1 (C-11), 38.3 (C-12), 138.5 (C-13), 121.3 (C-14), 29.3 (C-15), 16.3 (C-16), 107.3 (C-17), 33.5 (C-18), 22.4 (C-19), 15.2 (C-20), 128.3 (C-21), 147.8 (C-22), 116.3 (C-23), 113.6 (C-24), 149.5 (C-25), 116.5 (C-26), 171.1 (CH_3_CO), 21.6 (CH_3_CO). IR (cm^-1^): 3,401, 2,940, 1,701, 1,609, 1,501, 1,450, 1,367, 1,265, 1,199, 1,020, 958, 892, 753. M.S. (m/z, %): M^+^ 440 < 1%, 288 (9.0), 274 (11.8), 273 (55.6), 255 (31.8), 202 (14.2), 188 (11.4), 187 (34.1), 175 (21.3), 161 (14.0), 159 (13.5), 135 (100), 119 (36.9), 107 (45.9), 93 (42.1), 91 (26.7), 81 (29.6), 79 (24.6), 77 (11.4), 69 (21.4), 67 (19.1), 55 (21.1). 

*Synthesis of 2*β*-15-dihydroxy-ent-labda-8(17), 13-(Z)-diene* (**9**) *and 2*β*-15-dihydroxy-ent-labda-8(17), 13-(E)-diene* (**10**) *from* **5**-**6***mixture:* To a solution of **5**-**6** mixture (2.93g, 8.41 mmol) in MeOH (60 mL), finely divided K_2_CO_3_ (1.20g, 8.68 mmol) was added and the mixture stirred at room temperature for 0.5 h. After the TLC analysis indicated the completion of the reaction, the solvent was removed until a volume of approximately 5 mL remained and water (30 mL) was added, then 5% HCl (15 mL) was added, the mixture was extracted with EtOAc (3 x 30 mL) and the combined organic layers were washed successively with 10% NaHCO_3_ and water, dried over Na_2_SO_4_, filtered and evaporated. The crude (2.45 g) was redissolved in CH_2_Cl_2_ (10 mL) and chromatographed eluting with mixtures of petroleum ether/EtOAc of increasing polarity (19.8:0.2→12.2:7.8) to give three fractions.

Fraction I: compound **9** (0.292 g, 11.3%) colorless viscous oil, [α]_D_^23^ = -43.5º (c 0.96, CHCl_3_); ^1^H- NMR: 5.44 (bt, *J*= 7.1 Hz, 1H, H-14); 4.92 (s, 1H, H-17b); 4.61 (s, 1H, H-17a); 4.08 (dd, *J*= 7.1 and 2.5 Hz, 2H, H-15); 3.90 (ddt, *J*= 11.5, 11.5 and 4.2 Hz, 1H, H-2); 2.43 (ddd, *J*= 13.0, 4.2 and 2.5 Hz, 1H, H-7α); 2.09 (m, 3H, H-12a, H-12b and H-1α); 1.98 (dd, *J*= 13.0 and 5.1 Hz, 1H, H-7β); 1.77 (m, 1H, H-3α); 1.76 (s, 3H, H-16); 1.73 (m, 1H, H-6β); 1.63 (bd, *J*= 9.8 Hz, 1H, H-9); 1.61 (m, 1H, H-11b), 1.48 (m, 1H, H-11a); 1.31 (dd, *J*= 13.0 and 4.2 Hz, 1H, H-6α); 1.17 (dd, *J*= 11.5 and 11.5 Hz, 1H, H-3β); 1.08 (dd, *J*=12.5 and 2.7 Hz, 1H, H-5); 0.97 (dd, *J*= 11.5 and 11.5 Hz, 1H, H-1β); 0.91 (s, 3H, H-18); 0.86 (s, 3H, H-19); 0.73 (s, 3H, H-20). ^13^C-NMR: 48.0 (C-1), 65.6 (C-2), 51.0 (C-3), 35.0 (C-4), 54.8 (C-5), 23.9 (C-6), 38.0 (C-7), 147.7 (C-8), 55.7 (C-9), 40.9 (C-10), 21.9 (C-11), 30.4 (C-12), 140.3 (C-13), 124.6 (C-14), 59.0 (C-15), 23.4 (C-16), 107.3 (C-17), 33.6 (C-18), 22.6 (C-19), 15.4 (C-20). IR (cm^-1^): 3,380, 2,940, 1,641, 1,470, 1,445, 1,388, 1,035, 888, 754. M.S. (m/z, %): M^+^ 306 < 1%, 291 (12.8), 288 (12.8), 273 (57.2), 270 (13.4), 257 (12.9), 255 (50.2), 245 (13.3), 207 (19.9), 205 (18.5), 203 (14.1), 202 (15.1), 199 (12.9), 190 (13.4), 189 (20.5), 188 (13.4), 187 (40.9), 175 (36.1), 173 (17.8), 163 (14.3), 150 (21.5), 149 (18.5), 148 (13.5), 147 (43.6), 145 (25.0), 137 (18.7), 136 (19.7), 135 (100.0), 134 (27.1), 133 (44.0), 131 (21.8), 123 (28.5), 122 (24.1), 121 (68.7), 120 (32.0), 119 (57.5), 117 (16.6), 111 (13.9), 109 (47.8), 108 (22.2), 107 (84.9), 106 (19.4), 105 (59.3), 97 (23.8), 96 (14.5), 95 (67.5), 94 (22.1), 93 (96.2), 92 (14.3), 91 (59.0), 85 (20.4), 84 (23.2), 83 (27.7), 81 (71.2), 80 (16.8), 79 (61.5), 77 (32.6), 71 (25.0), 69 (61.9), 68 (16.9), 67 (47.5), 57 (41.6), 55 (59.0), 53 (24.1).

Fraction II: (0.527 g, 20.4%) viscous oil, mixture of **9**-**10**.

Fraction III: compound **10** (1.43 g, 55.4%) white needles, mp = 104.4-106.2°C (Et_2_O/MeOH), [α]_D_^23^ = -20.9º (c 1.05, CHCl_3_); ^1^H-NMR: 5.38 (bt, *J*= 6.9 Hz, 1H, H-14); 4.86 (s, 1H, H-17b); 4.54 (s, 1H, H-17a); 4.15 (d, *J*= 6.9 Hz, 2H, H-15); 3.88 (ddt, *J*= 12.0, 12.0 and 4.4 Hz, 1H, H-2); 2.40 (ddd, *J*= 12.6, 4.4 and 2.5 Hz, 1H, H-7α); 2.16 (ddd, *J*= 12.0, 9.5 and 4.2 Hz, 1H, H-12b); 2.10 (ddd, *J*= 11.6, 4.4 and 2.5 Hz, 1H, H-1α); 1.97 (ddd, *J*= 13.9, 12.6 and 5.1 Hz, 1H, H-7β); 1.84 (dd, *J*= 9.5 and 6.7 Hz, 1H, H-12a); 1.75 (m, 2H, H-3α and H-6β); 1.67 (s, 3H, H-16); 1.64 (m, 1H, H-11b); 1.63 (bd, *J*= 10.3 Hz, 1H, H-9); 1.49 (m, 1H, H-11a); 1.29 (dd, *J*= 12.6 and 4.2 Hz, 1H, H-6α); 1.15 (dd, *J*= 12.0 and 12.0 Hz, 1H, H-3β); 1.07 (dd, *J*=12.6 and 2.5 Hz, 1H, H-5); 0.96 (dd, *J*= 12.0 and 12.0 Hz, 1H, H-1β); 0.93 (s, 3H, H-18); 0.84 (s, 3H, H-19); 0.72 (s, 3H, H-20). ^13^C-NMR: 48.2 (C-1), 65.7 (C-2), 51.1 (C-3), 35.0 (C-4), 54.9 (C-5), 23.9 (C-6), 38.0 (C-7), 147.7 (C-8), 56.2 (C-9), 41.0 (C-10), 22.0 (C-11), 38.3 (C-12), 140.3 (C-13), 123.2 (C-14), 59.4 (C-15), 16.4 (C-16), 107.2 (C-17), 33.7 (C-18), 22.6 (C-19), 15.4 (C-20). IR (cm^-1^): 3,339, 2,935, 1646, 1,464, 1,440, 1,388, 1,363, 1,030, 888. M.S. (m/z, %): M^+^ 306 1.3%, 291 (22.7), 274 (14.1), 273 (68.2), 270 (11.8), 255 (48.3), 245 (15.1), 203 (13.9), 202 (18.4), 199 (11.9), 190 (14.4), 189 (13.2), 187 (41.2), 175 (33.8), 173 (13.2), 163 (12.4), 161 (27.0), 159 (19.3), 149 (13.3), 148 (13.2), 137 (14.5), 136 (18.3), 135 (100.0), 134 (20.6), 133 (38.5), 131 (19.4), 123 (24.4), 122 (19.3), 121 (62.7), 120 (31.9), 119 (44.1), 109 (42.9), 108 (17.9), 107 (73.9), 106 (13.9), 105 (45.0), 97 (15.8), 95 (50.2), 94 (16.1), 93 (77.6), 92 (11.8), 91 (44.7), 85 (13.4), 83 (20.5), 81 (58.3), 79 (48.9), 77 (23.9), 71 (18.0), 69 (51.2), 68 (14.9), 67 (37.9), 57 (32.7), 55 (42.4), 53 (18.2).

*Synthesis of 2β-hydroxy-15-phenyl-(22,25-dihydroxy)-ent-labda-8(17), 13(E)-diene* (**2**) *from* **9**: To a solution of 1,4-hydroquinone (0.086 g, 0.781 mmol) and BF_3_^.^THF (0.2 mL, 1.59 mmol) in freshly distilled 1,4-dioxane (5 mL) was slowly added dropwise with stirring at room temperature and under a N_2_ atmosphere, a solution of **9** (0.238 g, 0.777 mmol) in 1,4-dioxane (5 mL). After the addition was complete, stirring at room temperature and under N_2_ atmosphere was continued overnight. When the end of the reaction was verified by TLC, the mixture was poured onto crushed ice (app. 30 g) and the organic layer extracted with diethyl ether (2 x 30 mL), the ethereal layer was washed with 5% NaHCO_3_ (30 mL), then with water (2 x 20 mL) and dried over Na_2_SO_4_, filtered and evaporated. The crude was redissolved in CH_2_Cl_2_ (5 mL) and chromatographed on silica-gel with petroleum ether/EtOAc mixtures of increasing polarity (19.8:0.2→ 10.2:9.8). Two main fractions were obtained: Fraction I: 0.183 g of non-polar unidentified complex mixture (colorless viscous oil) and Fraction II:colorless viscous oil, 40 mg (13%) of compound **2**.

*Synthesis of* **2** *from* **10**: 1,4-hydroquinone (0.35 g, 3.18 mmol), BF_3_^.^THF (0.4 mL, 3.18 mmol) and **10** (0.897 g, 2.93 mmol) were used. After workup and CC purification: 0.420 g of complex mixture, 0.246 g (21%) of **2** and 0.172 g of unreacted 1,4-hydroqinone were obtained.

*Synthesis of* **2** *from* **9**-**10**: 1,4-hydroquinone (0.093 g, 0.85 mmol), BF_3_^.^THF (0.2 mL, 1.59 mmol) and **9**-**10** mixture (0.258 g, 0.85 mmol) were used. After workup and CC purification: 0.122 g of complex mixture, 0.057 g (17%) of diol **11** and 0.094 g (28%) of **2** were obtained.

Compound 11, colorless viscous oil, [α]_D_^23^ = -48.6º (c 0.14, CHCl_3_); ^1^H-NMR: 5.90 (dd, *J*= 17.4 and 10.8 Hz, 1H, H-14); 5.20 (dd, *J*= 17.4 and 1.2 Hz, 1H, H-15b); 5.06 (dd, *J*= 10.8 and 1.2 Hz, 1H, H-15a); 4.86 (s, 1H, H-17b); 4.56 (s, 1H, H-17a); 3.88 (ddt, *J*= 11.3, 11.3 and 4.4 Hz, 1H, H-2); 2.39 (ddd, *J*= 12.7, 4.4 and 2.5 Hz, 1H, H-7α); 2.10 (ddd, *J*= 11.3, 4.4 and 2.5 Hz, 1H, H-1α); 1.96 (ddd, *J*= 13.5, 12.7 and 5.4 Hz, 1H, H-7β); 1.75 (m, 3H, H-3α, H-12b and H-6β); 1.59 (m, 2H, H-11b and H-9); 1.38 (m, 1H, H-11a); 1.30 (m, 1H, H-12a); 1.27 (s, 3H, H-16); 1.25 (m, 1H, H-6α); 1.43 (dd, *J*= 11.0 and 11.0 Hz, 1H, H-3β); 1.07 (dd, *J*= 12.5 and 2.7 Hz, 1H, H-5); 0.97 (dd, *J*= 11.3 and 11.3 Hz, 1H, H-1β), 0.94 (s, 3H, H-18); 0.84 (s, 3H, H-19); 0.72 (s, 3H, H-20). ^13^C-NMR: 48.2 (C-1), 65.7 (C-2), 51.1 (C-3), 35.0 (C-4), 54.9 (C-5), 23.9 (C-6), 38.1 (C-7), 147.7 (C-8), 57.1 (C-9), 41.3 (C-10), 18.0 (C-11), 41.3 (C-12), 75.3 (C-13), 145.1 (C-14), 111.7 (C-15), 27.8 (C-16), 107.4 (C-17), 33.7 (C-18), 22.6 (C-19), 15.3 (C-20). IR (cm^-1^): 3,375, 2,935, 1,445, 1,368, 1,035, 887. M.S. (m/z, %): M^+^ 306 < 1%, 273 (26.5), 260 (18.1), 255 (50.8), 202 (20.7), 201 (17.9), 189 (18.2), 188 (21.0), 187 (46.8), 175 (30.6), 173 (17.4), 161 (21.1), 159 (18.8), 145 (18.8), 137 (18.0), 136 (18.6), 135 (100.0), 134 (30.9), 133 (30.8), 123 (17.4), 122 (16.8), 121 (49.6), 120 (29.7), 119 (36.9), 109 (32.1), 108 (14.4), 107 (66.1), 105 (36.7), 95 (45.0), 94 (24.5), 93 (73.8), 91 (37.4), 83 (16.5), 81 (47.8), 80 (22.3), 79 (43.1), 77 (18.5), 71 (49.7), 69 (40.9), 67 (33.2), 57 (23.8), 55 (41.0). 

Compound 2, colorless viscous oil, [α]_D_^23^ = -18.1º (c 0.38, CHCl_3_); ^1^H-NMR: 6.67 (d, *J*= 8.6 Hz, 1H, H-23); 6.64 (d, *J*= 2.9 Hz, 1H, H-26); 6.57 (dd, *J*= 8.6 and 2.9 Hz, 1H, H-24); 5.28 (t, *J*= 7.1 Hz, 1H, H-14); 4.84 (s, 1H, H-17b); 4.54 (s, 1H, H-17a); 3.91 (ddt, *J*= 12.0, 12.0 and 4.2 Hz, 1H, H-2); 3.29 (d, *J*= 7.1 Hz, 2H, H-15); 2.37 (ddd, *J*= 12.0, 4.7 and 2.2 Hz, 1H, H-7α); 2.17 (m, 1H, H-12b); 2.10 (m, 1H, H-1α); 1.89 (ddd, *J*= 15.2, 12.7 and 4.4 Hz, 1H, H-7β); 1.83 (m, 1H, H-12a); 1.75 (m, 1H, H-3α); 1.72 (s, 3H, H-16); 1.68 (m, 1H, H-6β); 1.60 (bd, *J*= 11.3 Hz, 1H, H-9); 1.55 (m, 2H, H-11a and H-11b); 1.26 (dd, *J*= 11.5 and 3.9 Hz, 1H, H-6α); 1.17 (dd, *J*= 12.0 and 12.0 Hz, 1H, H-3β); 0.97 (dd, *J*= 12.7 and 2.5 Hz, 1H, H-5); 0.94 (dd, *J*= 12.0 and 12.0 Hz, 1H, H-1β); 0.91 (s, 3H, H-18); 0.82 (s, 3H, H-19); 0.69 (s, 3H, H-20). ^13^C-NMR: 47.9 (C-1), 66.1 (C-2), 50.7 (C-3), 35.0 (C-4), 54.6 (C-5), 23.8 (C-6), 38.0 (C-7), 147.6 (C-8), 55.3 (C-9), 40.8 (C-10), 22.0 (C-11), 37.7 (C-12), 138.1 (C-13), 121.7 (C-14), 29.0 (C-15), 16.8 (C-16), 107.2 (C-17), 33.6 (C-18), 22.6 (C-19), 15.3 (C-20), 128.7 (C-21), 147.7 (C-22), 116.3 (C-23), 113.7 (C-24), 149.5 (C-25), 116.6 (C-26). IR (cm^-1^): 3,375, 2,935, 1,455, 1,199, 1,025. M.S. (m/z, %): M^+^ 398 < 1%, 280 (2.6), 279 (14.5), 168 (3.3), 167 (36.4), 149 (100.0), 113 (8.4), 112 (5.3), 104 (5.5), 84 (2.8), 83 (5.1), 76 (2.7), 71 (13.8), 70 (12.2), 69 (33.0), 57 (17.8), 55 (7.0).
